# Carbon dioxide enhances *Akkermansia muciniphila* fitness and anti-obesity efficacy in high-fat diet mice

**DOI:** 10.1093/ismejo/wraf034

**Published:** 2025-02-23

**Authors:** Xiangfeng Wang, Qianqian Yang, Changping Shi, Yuyang Wang, Dingming Guo, Xuchun Wan, Pengyuan Dong, Qianyao Zhang, Yueyan Hu, Ruilin Zhang, Hongju Yang, Weihua Chen, Zhi Liu

**Affiliations:** Key Laboratory of Molecular Biophysics of the Ministry of Education, Hubei Key Laboratory of Bioinformatics and Molecular Imaging, Department of Biotechnology, College of Life Science and Technology, Huazhong University of Science and Technology, Luoyu Road 1037, Hongshan District, Wuhan, Hubei 430074, China; Key Laboratory of Molecular Biophysics of the Ministry of Education, Hubei Key Laboratory of Bioinformatics and Molecular Imaging, Department of Biotechnology, College of Life Science and Technology, Huazhong University of Science and Technology, Luoyu Road 1037, Hongshan District, Wuhan, Hubei 430074, China; Key Laboratory of Molecular Biophysics of the Ministry of Education, Hubei Key Laboratory of Bioinformatics and Molecular Imaging, Department of Biotechnology, College of Life Science and Technology, Huazhong University of Science and Technology, Luoyu Road 1037, Hongshan District, Wuhan, Hubei 430074, China; Key Laboratory of Molecular Biophysics of the Ministry of Education, Hubei Key Laboratory of Bioinformatics and Molecular Imaging, Department of Biotechnology, College of Life Science and Technology, Huazhong University of Science and Technology, Luoyu Road 1037, Hongshan District, Wuhan, Hubei 430074, China; Key Laboratory of Molecular Biophysics of the Ministry of Education, Hubei Key Laboratory of Bioinformatics and Molecular Imaging, Department of Biotechnology, College of Life Science and Technology, Huazhong University of Science and Technology, Luoyu Road 1037, Hongshan District, Wuhan, Hubei 430074, China; Key Laboratory of Molecular Biophysics of the Ministry of Education, Hubei Key Laboratory of Bioinformatics and Molecular Imaging, Department of Biotechnology, College of Life Science and Technology, Huazhong University of Science and Technology, Luoyu Road 1037, Hongshan District, Wuhan, Hubei 430074, China; Xi’an Jiaotong-Liverpool University, 111 Ren’ai Road, Suzhou Industrial Park, Suzhou, Jiangsu 215123, China; NHC Key Laboratory of Drug Addiction Medicine, School of Forensic Medicine, 1168 West Chunrong Road, Yuhua Avenue, Chenggong District, Kunming Medical University, Kunming 650500, China; Division of geriatric Gastroenterology, The First Affiliated Hospital of Kunming Medical University, Xichang Road No. 153, Wuhua District, Kunming, Yunnan 650032, China; NHC Key Laboratory of Drug Addiction Medicine, School of Forensic Medicine, 1168 West Chunrong Road, Yuhua Avenue, Chenggong District, Kunming Medical University, Kunming 650500, China; Division of geriatric Gastroenterology, The First Affiliated Hospital of Kunming Medical University, Xichang Road No. 153, Wuhua District, Kunming, Yunnan 650032, China; Key Laboratory of Molecular Biophysics of the Ministry of Education, Hubei Key Laboratory of Bioinformatics and Molecular Imaging, Center for Artificial Intelligence Biology, Department of Bioinformatics and Systems Biology, College of Life Science and Technology, Huazhong University of Science and Technology, Luoyu Road 1037, Hongshan District, Wuhan, Hubei 430074, China; Key Laboratory of Molecular Biophysics of the Ministry of Education, Hubei Key Laboratory of Bioinformatics and Molecular Imaging, Department of Biotechnology, College of Life Science and Technology, Huazhong University of Science and Technology, Luoyu Road 1037, Hongshan District, Wuhan, Hubei 430074, China

**Keywords:** *A. muciniphila*, carbon dioxide, live bacterial delivery system, bacterial growth, obesity

## Abstract

Numerous studies and clinical applications have underscored the therapeutic potential of the indigenous gut bacterium *Akkermansia muciniphila* in various diseases. However, our understanding of how *Akkermansia muciniphila* senses and responds to host gastrointestinal signals remains limited. Here, we demonstrate that *A. muciniphila* exhibits rapid growth, facilitated by its self-produced carbon dioxide (CO₂), with key enzymes such as glutamate decarboxylase, carbonic anhydrase, and pyruvate ferredoxin oxidoreductase playing pivotal roles. Additionally, we design a novel delivery system, comprising calcium carbonate, inulin, *A. muciniphila*, and sodium alginate, which enhances *A. muciniphila* growth and facilitates the expression of part probiotic genes in mice intestinal milieu. Notably, the administration of this delivery system induces weight loss in mice fed high-fat diets. Furthermore, we elucidate the significant impact of CO₂ on the composition and functional genes of the human gut microbiota, with genes encoding carbonic anhydrase and amino acid metabolism enzymes exhibiting heightened responsiveness. These findings reveal a novel mechanism by which gut commensal bacteria sense and respond to gaseous molecules, thereby promoting growth. Moreover, they suggest the potential for designing rational therapeutic strategies utilizing live bacterial delivery systems to enhance probiotic growth and ameliorate gut microbiota-related diseases.

## Introduction

The human gastrointestinal tract harbors a vast and diverse microbiota that significantly influences various aspects of human health, including metabolic syndrome [1], chronic intestinal inflammation [2], cancer [3], cardiovascular diseases [4], and neurological disorders [5]. The composition and activities of the gut microbiota are affected by a multitude of factors, such as antibiotics [6], surgical treatments [7], diet [8], lifestyle [9], and environmental conditions [10]. Clinical trials have demonstrated that strategies targeting gut microbiota modification using prebiotics [11], trace elements, and small molecule drugs can effectively improve disease treatment outcomes [12].

The mechanisms by which gut microbiota exert their physiological functions are complex, involving a diverse array of microorganisms and the production of various gases, including carbon dioxide (CO₂), hydrogen [13], methane [14], and hydrogen sulfide (H₂S) [15]. These volatile intestinal gases, due to their low molecular weight and high diffusivity, can significantly influence the growth and activity of gut microbiota [16]. Moreover, they can reach various tissues and exert systemic effects on human health and disease through the bloodstream and other body fluid circulation systems. For instance, gut microbiota can digest dietary choline to produce gaseous trimethylamine, which exacerbates atherosclerosis [17] and disrupts hippocampal ultrastructure in mice [18].

In clinical diagnostics, measurements of hydrogen and methane in exhaled breath is widely utilized as biomarkers for various diseases, such as lactose intolerance, small intestinal bacterial overgrowth, and irritable bowel syndrome (IBS) [19]. Intestinal bacteria, including genera such as *Ruminococcus*, *Bacteroides* and *Clostridium*, are capable of producing hydrogen via enzymatic pathways [20,21]. The hydrogen gas thus generated can subsequently be converted into methane and H₂S by methanogenic archaea and sulfate-reducing bacteria, respectively. H₂S, as a gaseous signaling molecule, can be utilized in the treatment of colitis by mitigating dysbiosis and aiding in the reconstruction of the mucus layer, as well as playing a role in blood pressure regulation [22,23]. However, at high concentrations, H₂S is potentially toxic to human tissues, particularly when co-present with nitric oxide (NO), impairing various physiological processes [24].

Like NO and H₂S, carbon monoxide (CO) also exhibits vasodilatory and cardioprotective effects. CO is produced in reactions catalyzed by heme oxygenase, and it has been reported that gut microbiota also express heme oxygenase homologs [25]. Additionally, large amounts of ammonia (NH₃) are produced by various bacteria in the mammalian gut, with hyperammonemia being associated with liver disease and neurotoxic effects [26].

Despite its low concentration in the atmosphere (0.04%, v/v), CO₂ is the primary gas [27] (9.9%, v/v) produced during the bacterial fermentation of carbohydrates in the distal small intestine and colon [27]. CO₂ plays a crucial role in gut microbial growth and activity. It serves as an important factor in pH homeostasis in cells [28] and participates in various metabolic processes, including amino acid and nucleotide biosynthesis [29], cyanate detoxification [30], and cellular physiology regulation [31]. Investigating the interplay between CO_2_ and gut microbiota holds promise for enhancing our understanding and management of numerous gastrointestinal disorders.

Among the gut microbiota, *Akkermansia muciniphila* ([32]) is a Gram-negative bacterium that utilizes mucin as its main carbon and nitrogen source [33]. *A. muciniphila* has been implicated in improving intestinal inflammation [34], metabolic syndrome [35], the immune response to tumors [36], and nervous system diseases [37,38]. The outer membrane proteins of *A. muciniphila*, such as muc_1100 [34] and P9 [39] (Amuc_1831), play roles in regulating various physiological processes. Additionally, *A. muciniphila* has shown efficacy in modulating the intestinal immune microenvironment and enhancing the efficacy of programmed cell death ligand 1 (PD-L1) therapy in non-small cell lung cancer [40].

In this study, we elucidate the role of CO₂ in promoting the growth of *A. muciniphila* and develop a novel delivery system based on calcium carbonate and inulin (CIAs) to enhance *A. muciniphila* abundance in a high-fat diet-induced obesity mouse model. Furthermore, we elucidate the significant influence of CO₂ on the composition and functional genes of the human gut microbiota, with genes encoding carbonic anhydrase and amino acid metabolism enzymes exhibiting heightened responsiveness. Our findings shed light on the potential therapeutic applications of *A. muciniphila* and highlight the importance of CO₂ in modulating gut microbiota composition and function.

## Materials and methods

### Bacterial culture

The *A. muciniphila* strain (ATCC BAA-835) used in this study was purchased from BENAGEN (Wuhan, China). *A. muciniphila* was cultured in BHIM (Brain-Heart-Infusion-Mucin) medium in both liquid and solid forms. BHIM medium was prepared according to the manufacturer’s instruction: For 1 L of BHIM, brain heart infusion (BHI, 37 g, OXOID), yeast extract (5 g, OXOID) and 1 ml 0.1% resazurin (Sigma) are dissolved in pure water (850 ml), stirring and purging with anaerobic nitrogen gas and heated for deoxygenation. After deoxygenation, 200 ml 1.25% mucin solution (Sigma) and 1 g cysteine hydrochloride (Sangon, Shanghai China) was added. The deoxygenated medium was then sealed in anaerobic bottles with butyl rubber stoppers and autoclaved at 121°C for 30 minutes. For solid plate cultures, 1.5% agar powder was added before sealing the medium into the anaerobic bottle.

The gas conditions of the anaerobic culture environment are 90% N_2_, 10% H_2_, and the corresponding CO_2_ environment is 80% N_2_, 10% H_2_, 10% CO_2_. A 1:100 solution of *A. muciniphila* was inoculated onto 5 ml of fresh BHIM medium and incubated at 37°C anaerobically (Coy Laboratory Products) for 48 h.

### Pre-treatment of RNA and protein sample

For the Treatment group, CO_2_ was rapidly removed from the culture medium by magnetic stirring with the lid open. For the Control group, the lid was tightened to allow CO_2_ to accumulate in the culture medium. Bacterial growth was monitored by measuring OD600 at different time points. We collected samples from the Treatment group, both before magnetic stirring (18 hours) and after 6 hours of stirring (24 hours), for transcriptomic and proteomic analysis.

RNA samples were extracted according to the manufacturer’s instruction (RNA extraction kit, Qiagen). Transcriptome sequencing was then submitted (Majorbio, Shanghai China). Bacterial solution samples were added to the lysis buffer (Qiagen), and centrifuged at 8000 rpm for 2 min to remove the sample supernatant, with 50 μg of sample per well (control: BCA, ThermoFisher Scientific). Protein was checked by SDS-PAGE. Protein electrophoresis was performed at 80 V. The protein gel was stained with Coomassie Brilliant Blue (Sangon, Shanghai China), and agitated at room temperature for 5 minutes, Gel destaining was then performed at room temperature with destaining solution (Sangon, Shanghai China). The acquired samples were then submitted for proteomic sequencing.

### Transcriptome and proteome data analysis

The quality of the data obtained from transcriptome sequencing was assessed using FastQC (https://github.com/s-andrews/FastQC). Hisat2 [41] was used to align clean data to the reference genome of *A. muciniphila* (GCF_009731575.1, https://www.ncbi.nlm.nih.gov/datasets/genome/?taxon=239935). The output data was converted to bam format using samtools [42]. For the transcriptomic data, the Stringtie [43] tool is used to assemble transcripts and calculate gene expression, which provided the expression levels of each gene. Similarly, for the proteomic data, expression levels for each protein and its corresponding encoding gene was obtained using Uniprot [44] database. The two datasets were integrated by matching gene IDs shared by the transcriptomic and proteomics datasets (Supplemental Tables S1 and S2). By querying the genes of *A. muciniphila* ATCC BAA-835 in the BioCyc database, identified related enzyme gene encoding identified the CO_2_-related genes. This subset of genes (https://www.biocyc.org/compound?orgid=AMUC349741&id=CARBON-DIOXIDE#RXNS, Supplementary Table S3) was selected and changes in gene expression were detected in transcriptomic and proteomic datasets. After obtaining RNA count matrix and protein quantitative expression matrix, RNA count matrix and protein quantitative expression matrix were filtered respectively, and genes and proteins whose mean value was less than or equal to 1 were filtered out. In the RNA-seq data, the DESeq2 R package was used to calculate the fold change and significance of each gene in the two groups. The proteome data converted the original protein quantitative value as follows: value-new = log10(value-old+1). The limma R package was used to calculate the fold change and significance of each protein in the two groups. GO enrichment analysis was performed by using cluster Profiler R package. The analysis results were visualized using the ggplot2 R package.

## Cultureomics of human fecal samples

### Medium preparation and bacterial culture

Fecal samples were collected from five healthy volunteers (informed consent was obtained from the volunteers for sample acquisition and analysis) for subsequent processing. This sample was then cultured under two conditions: with and without the addition of CO_2_, across each of the culture media. For liquid culture media, fecal microbiota was inoculated into 2 ml of liquid medium and incubated under anaerobic conditions at 37°C for 14 days, with or without CO_2_. DNA was extracted using a DNA extraction kit, and the concentration of the DNA was adjusted to uniform levels. 30 μl from each culture medium was combined to form a 300 μl final sample. For solid culture media, fecal microbiota were subjected to gradient dilution [10–1 to 10–6]. 100 μl of the 10^−1^, 10^−3^, 10^−5^, and 10^−6^ dilutions were plated on agar plates and incubated under anaerobic conditions at 37°C, with or without CO_2_. On Day 7, colonies were collected from the 10^−1^ dilution plate; on Day 10, from the 10^−3^ dilution plate; and on Day 14, from the 10^−5^ and 10^−6^ dilution plates. The colonies from the 10^−5^ and 10^−6^ dilution plates were combined into a single sample. DNA was extracted using a DNA extraction kit, and the DNA concentration was adjusted to uniform levels. 20 μl was taken from each dilution, with a total of 60 μl collected from each culture medium, and all samples were combined into a 300 μl final sample. In total, including the fecal sample, we obtained 5 sequencing samples. The extracted DNA was frozen at −80°C. PE150 sequencing was performed by Shenzhen Microservice Technology Co, LTD.

### Metagenomic sequencing data analysis

For all metagenomic sequencing data, fastp was used to filter out low-quality sequences and adapter sequences [45]. To remove host sequences from the fecal samples, the human reference genome (GRCh38.p14) was downloaded from GENCODE (https://www.gencodegenes.org/) and an index was built using the bowtie2-build command. The KneadData (https://github.com/biobakery/kneaddata) tool was then used to remove host sequences. Clean reads after quality control were used to calculate the relative abundance of microbial species using MetaPhlAn 3.0 [46]. To characterize the general metabolic and functional composition of our metagenomic sequencing data, the clean reads was analyzed by using HUMAnN 3.0 [46]. Data with species-level relative abundance less than 0.01% were filtered out for subsequent analysis. The average relative abundance of each species under CO_2_+ or CO_2_- conditions was calculated as the relative abundance for each group. The VennDiagram R package was used to analyze and visualize the overlap of species among fecal, CO_2_+, and CO_2_- groups. Statistical differences in genes and metabolic pathways between CO_2_+ and CO_2_- groups were compared using Student’s T-test.

### Enzyme inhibition experiments

According to relevant literature [47–49], the corresponding enzyme inhibitors of glutamate dehydrogenase, carbonic anhydrase and pyruvate ferredoxin oxidoreductase are L-malic acid, Acetazolamide and Nitazoxanide. Inhibitors were purchased from Sinopharm Chemical Reagent LTD. The inhibitor group was added with 0.01 mg/ml enzyme inhibitor, and the CO_2_ group was added with 5% (v/v) CO_2_ in addition to inhibitor solution. All groups were cultured at 37°C for 48 hours. OD600 is detected and converted to colony-forming units (CFU) for counting.

### Quantitative PCR

For detection of gene expression in *A. muciniphila*, reverse transcription quantitative PCR (RT-qPCR) was used. *A. muciniphila* was grown in BHIM medium to OD = 0.3. Total RNA was extracted using the bacterial RNA extraction kit (RN-01, Aidlab) according to the method provided by the manufacturer. About 500 ng of total RNA was used for gDNA digestion using the HiScript II 1 st Strand cDNA Synthesis Kit (R212, Vazyme), and the first strand cDNA was synthesized with the specific primer (T2) corresponding to the gene. Each qPCR reaction system (20 μl) consisted of 10 μl 2 × T5 Fast qPCR Mix (SYBR Green I) (TSE202, TSINGKE), 7.4 μl H_2_O, 0.8 μl 10 μM forward primer, 0.8 μl 10 μM reverse primer and 1 μl 10-time diluted reverse transcription system. All primers were tested for PCR specificity and efficiency. The 16S rRNA gene was used for normalization. Amplification using the CFX Connect Real time Inspection System (Bio-Rad) was conducted as follows: After the initial denaturation of 95 °C for 1 min, 40 amplification cycles were performed, including denaturation of 95 °C for 10 s, annealing of 55 °C for 5 s, extension of 72 °C for 15 s, and finally melting curves were generated. The experiment consisted of three biological replicates and two detection replicates. Results are expressed as average values, and error bars represent standard deviations.

### CIAs preparation

Culture broth containing *A. muciniphila* cells in the stable phase was added to different tubes, with the volume of the solution in each tube not exceeding 25 ml. The solution was centrifuged at 8000 rpm and 4 °C for 20 min. After centrifugation, the supernatant was discarded. The *A. muciniphila* cells at the bottom of the tube was resuspended with pure water, and centrifugation was repeated under the same conditions. The supernatant was discarded again. Finally, the solution in each test tube was concentrated to 5 ml. 50 μl of 0.15% (w/w) calcium chloride solution was added to the concentrated *A. muciniphila* solution and the solution was vortexed for 1 min. Then, a mixture of sodium alginate (4% w/v) and inulin (4% w/v), 5 ml in total, was added. The solution was then vortexed for 3 min. After mixing, the mixture was drawn into a 5 ml syringe, and the syringe needle connection was sealed with a sealing film. The syringe was then transferred to an electrostatic spray device. The syringe needle was replaced, and a beaker containing 200 ml of calcium chloride fixative (0.15% w/w) was placed in the center of the electrostatic spray device. The distance between the electrospray needle and the beaker was adjusted to 20 cm, and the electrostatic spray voltage was set to 12 kV with an injection speed of 0.75 ml per min. The curing process was started. After curing, the mixture was transferred to a new beaker for 2 hours of solidification. The upper calcium chloride solution was discarded, and ultrapure water was added to resuspend the CIAs. After allowing the CIAs in the system to precipitate, the supernatant was discarded, and ultrapure water was added for washing. This washing process was repeated 20 times to reduce the calcium ion concentration in the liquid. After washing, the CIAs were resuspended in a freeze-drying protective agent (Supplementary Materials), placed in a constant temperature incubator shaker (37°C, 200 rpm) for 30 min, and then left at room temperature for 10 min. Once CIAs sank to the bottom of the tube, the top solution was removed. CIAs were pre-frozen at −20°C for 3 h, then transferred to a pre-cooled freeze dryer (LABCONCO FreeZone®) and freeze-dried for 72 h. Finally, CIAs were stored at 4°C.

### Simulated in vitro digestion of CIAs

Simulated gastric fluid: Add 10% hydrochloric acid to 1 L of ultrapure water and adjust to pH = 2. Then, add 10 g of pepsin, mix thoroughly, filter through 0.22 μm membrane filter to remove bacteria, then place in refrigerator at 4°C for cold storage, and then preheat to 37°C before use. Simulated small intestine solution: weigh 6.8 g of dipotassium hydrogen phosphate, add to 1 L of ultrapure water, use 10% sodium hydroxide solution to adjust the solution to pH = 6.8, add 10 g of trypsin, stir well, filter through a 0.22 μm membrane to remove bacteria, place in a refrigerator at 4°C, and preheat to 37°C before use. During the 0–3-hour period, both groups were incubated simultaneously in simulated gastric fluid. In the 3–8-hour period, the samples that had been incubated in simulated gastric fluid were transferred to simulated intestinal fluid for further incubation. The entire incubation process was conducted at 37°C.

### Animal experiment

6-week-old male C57BL/6 mice were purchased from Hubei Province Center for Disease Control and Prevention (Wuhan, China). All mice were housed in a specific pathogen-free environment at a constant temperature (22 ± 3°C), with a 12-hour light/dark cycle. All mice were allowed to acclimatize for 1 week after arrival. During the experiments, all mice had free access to water and food.

During the experiments, mice were fed either a control diet (SYSE BIO, China) or a 60% kcal high-fat diet (SYSE BIO, China, Table S4). Mice that exceeded normal weight by at least 30% were considered obese.

All mice were given a water fast for 3 hours before intragastric administration, followed by direct intragastric administration. In the *A. muciniphila* (AKK) and CIAs group, 5 × 10^7^ CFU of *A. muciniphila* (CIAs were converted according to living bacteria counts) were suspended in 200 μl of buffer. The AKK group was intragastrical injected with *A. muciniphila* and PBS buffer, the CIAs group was intragastrical injected with CIAs and water. The PBS group is treated with 200 μl of PBS buffer. The mice in each group resumed eating and drinking 2 hours after gavage. Gavage was conducted every 3 days for 66 days.

The bodyweight and food intake of mice were recorded weekly. The mice were sacrificed at the end of the experiments after overnight fasting. Blood was immediately collected from the animals and allowed to clot for 30 min at room temperature. Serum was separated by centrifuging the blood samples at 3000 rpm for 15 min and finally stored at −80°C until use. Unilateral liver and gonadal adipose tissue were excised rapidly, washed in ice-cold PBS (pH = 7.4), blotted and weighed. Then cut a part of the liver and gonadal adipose tissues were cut and immersed in 4% paraformaldehyde fixative for fixation and hematoxylin and eosin (H&E) staining. The tissues after fixation were embedded in paraffin and were sectioned at 5 μm thickness sections. After staining, sections were captured at 200× magnification using a Nikon Digital Imaging System. Finally, the results are quantified by ImageJ software.

Blood index assay was performed using the kit provided by Nanjing Jiancheng Bioengineering Institute. The results were quantified and calculated based on the corresponding standard curve, including total cholesterol (TC, A111–1-1), triacylglycerol (TG, A110–1-1), low-density lipoprotein cholesterol (LDL-C, A113–1-1), high-density lipoprotein cholesterol (HDL-C, A112–1-1), and Lipopolysaccharides (LPS, A039–1-1).

### Statistical data processing

Statistical analysis was performed using SPSS 25.0 software (SPSS Inc) or R. Charts are drawn by GraphPad Prism version 9.2.0 (GraphPad Software). or R. *P* < 0.05 is considered statistically significant, otherwise there is no statistical significance (ns). The confidence levels in all charts are indicated by ^*^(*P* < 0.05), ^**^(*P* < 0.01), ^***^(*P* < 0.001), ^****^(*P* < 0.0001). Continuous variables are expressed as mean ± standard deviation, and categorical variables are expressed as absolute frequency and relative frequency. Student’s T-test or Mann–Whitney U test were used for differences in continuous variables, and Chi-square test or Fisher exact test were used for categorical variables. All statistical tests are bilateral tests.

## Results

### Self-produced CO₂ significantly enhanced *A. muciniphila* growth

**Figure 1:**
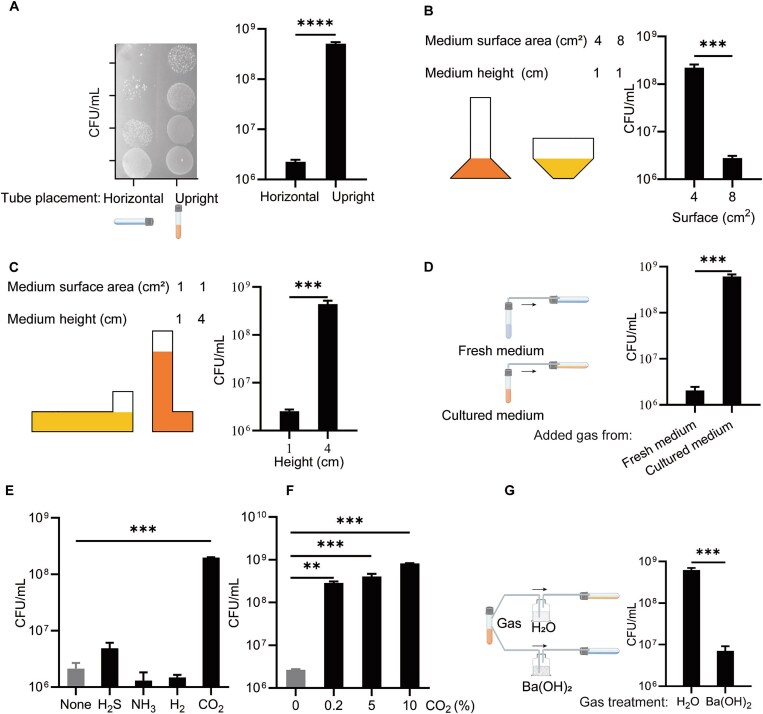
*A. muciniphila* self-produced CO_2_ triggers its quick growth. Growth of *A. muciniphila* under anaerobic test tube placement (A), different liquid surface areas (B), different liquid column heights (C), addition of overhead gas from culture medium (D), major volatile gases produced by *A. muciniphila* as inferred by GSMM (E), different concentrations of CO_2_ (F), and overhead gas from culture medium treated with Ba(OH)_2_ (G). ^**^P < 0.01, ^***^P < 0.001, ^****^P < 0.0001.

We utilized anaerobic tubes sealed with butyl rubber stoppers and aluminum caps to culture *A. muciniphila*. The culture conditions included 3 ml of BHIM (Brain-Heart-Infusion Mucin) medium inoculated with a 5% overnight culture of *A. muciniphila*, incubated statically at 37°C. After 48 hours of incubation, we observed that the bacterial growth was normal when the tubes were positioned vertically, whereas the culture remained clear when the tubes were positioned horizontally. Gradient dilution combined with plate count results indicated a 1000-fold difference in CFU between the two conditions (Fig. 1A).

Given that the differences between the two culture conditions were solely attributed to the surface area of the culture medium and the height of the liquid column, we employed 3D printing technology to create culture vessels with varying surface areas (Fig. 1B left, Fig. S1A) and liquid column heights (Fig. 1C left, Fig. S1B) for the cultivation of *A. muciniphila*. The results revealed that both surface area and liquid column height significantly impacted the growth of *A. muciniphila* (Fig. 1B, C). A smaller surface area and higher liquid column height likely reduced the evaporation rate of gas molecules, leading us to hypothesize that volatile small molecules produced during *A. muciniphila* cultivation regulate its growth rate. This hypothesis was confirmed by experiments where the addition of headspace gas from overnight cultures stimulated the rapid growth of *A. muciniphila* under horizontal conditions (Fig. 1D).

A metabolic network analysis of the *A. muciniphila* genome suggested that the volatile small molecules produced could include hydrogen sulfide (H_2_S), ammonia (NH_3_), hydrogen (H_2_), and CO₂ (Fig. S2). Among these, only CO₂ significantly stimulated *A. muciniphila* growth in a concentration-dependent manner (Fig. 1E, F, Fig. S3). Further investigation revealed that headspace gas from overnight cultures treated with barium hydroxide lost the ability to stimulate *A. muciniphila* growth, indicating that *A. muciniphila* promotes its rapid growth through self-produced CO₂ (Fig. 1G).

**Figure 2:**
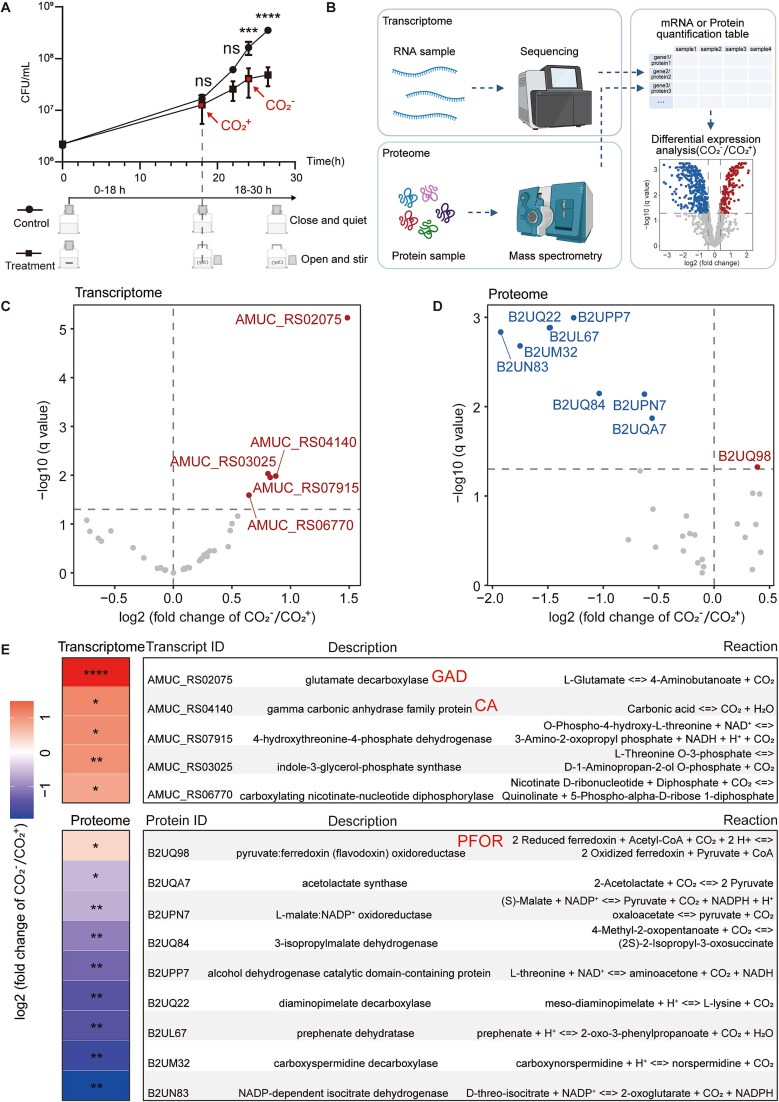
Transcriptome and Proteome of *A. muciniphila* under different CO_2_ conditions. (A) The impact of CO_2_ depletion on *A. muciniphila* growth and a schematic diagram of sampling points for omics analysis. (B) Schematic diagram of *A. muciniphila* transcriptome and proteome analysis. Volcano plot of transcriptome data (C) and proteome data (D) for CO_2_-related genes. (E) Information on CO_2_-related genes with significant differences in the transcriptome and proteome. Graph A: ^***^P < 0.001, ^****^P < 0.0001, ns (no significance); Graph E: ^*^, q-value (adjusted *P*-value) <0.05, ^**^q < 0.01, ^****^q < 0.0001.

### Key enzymes and pathways in *a. muciniphila* CO_2_-dependent growth

**Figure 3:**
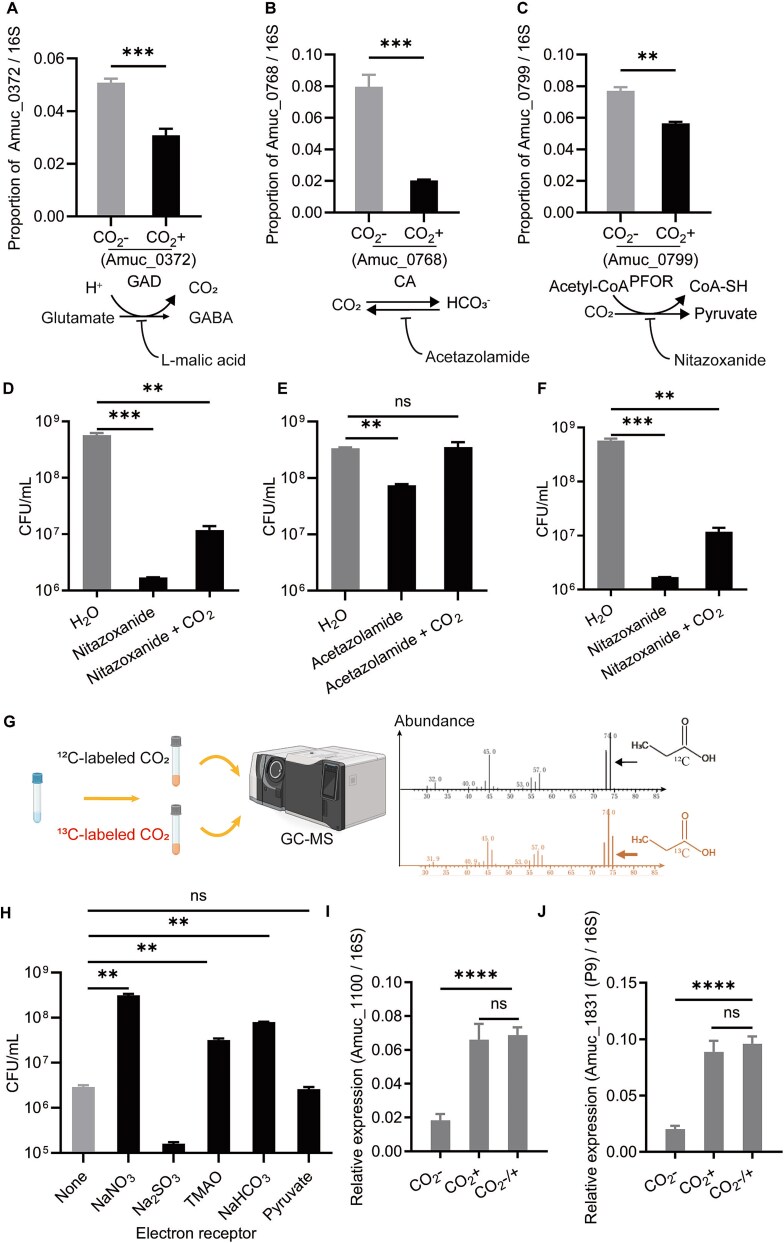
Key genes and pathways in CO_2_-regulated growth and beneficial effects of *A. muciniphila*. Differential expression of CO_2_-related genes in *A. muciniphila*. Including CO_2_ production (A, GAD), fixation (B, CA), and metabolism (C, PFOR). Inhibition of the activities of these genes affects *A. muciniphila* CO_2_-dependent growth, and additional CO_2_ supplementation growth phenotype. (D) L-malic acid inhibits GAD, (E) Acetazolamide inhibits CA, (F) Nitazoxanide inhibits PFOR. (G) CG-MS detected ^13^C-labeled propionic acid by adding ^13^CO_2_ to *A. muciniphila* culture environment. (H) Results of adding different electron acceptors on the growth of *A. muciniphila*. CO_2_ induces the expression of Amuc_1100 (I) and Amuc_1831 (J) in *A. muciniphila*. ^**^P < 0.01, ^***^P < 0.001, ^****^P < 0.0001, ns (no significance).

To elucidate the molecular mechanisms by which CO₂ promotes the growth of *A. muciniphila*, we prepared anaerobic air devoid of CO₂ (N₂ 90%, H₂ 10%). Under these conditions, the CO₂ produced and accumulated by *A. muciniphila* in the culture medium could be rapidly released into the anaerobic chamber by uncovering and stirring. We observed that the growth rate of *A. muciniphila*, which was initially normal, significantly decreased after rapid stirring with a magnetic stirrer (Fig. 2A), further confirming that *A. muciniphila* growth depends on dissolved CO₂. We selected *A. muciniphila* cells from normal growth conditions (Fig. 2A, labeled as CO₂+) and CO_2_-devoid growth conditions (Fig. 2A, labeled as CO₂-) for transcriptomic and proteomic analysis (Fig. 2B). Using the BioCyc [50] database, we identified 36 genes in *A. muciniphila* that are directly related to CO₂ (Table S3). We selected this gene subset and examined the changes in gene expression in both our transcriptomic and proteomic datasets. We found that AMUC_RS02075 (Amuc_0372, glutamate decarboxylase) and AMUC_RS04140 (Amuc_0768, gamma carbonic anhydrase family protein) showed the largest fold changes (CO_2_-/CO_2_+) in the transcriptomic data, while B2UQ98 (pyruvate ferredoxin oxidoreductase, PFOR) had the largest fold change (CO_2_-/CO_2_+) in the proteomic data. Therefore, we chose these three enzymes for further analysis. The proteomic data indicated that pyruvate ferredoxin oxidoreductase (PFOR), which catalyzes the conversion of CO₂ and acetyl-CoA to pyruvate, was also significantly upregulated following CO₂ dissipation (Fig. 2C-E; Table S5, S6). These findings were validated through RT-PCR experiments (Fig. 3A-C). Inhibitors of these three enzymes significantly suppressed the growth of *A. muciniphila* under normal culture conditions (Ф18 × 150 mm anaerobic tubes, 3 ml BHIM medium, 5% inoculum, 37°C static incubation), but the addition of CO₂ effectively mitigated these inhibitory effects (Fig. 3D-F). These results indicate that *A. muciniphila* may produce CO₂ via GAD and convert CO₂ to the more soluble HCO₃^−^ through CA, thereby increasing intracellular concentrations. PFOR is a key enzyme in the reductive tricarboxylic acid (rTCA) cycle, suggests that *A. muciniphila* fixes CO₂ through the rTCA cycle. Using ^13^CO₂ addition experiment, we detected ^13^C-labeled propanoic acid in the products, confirming that *A. muciniphila* utilizes CO₂ to produce short-chain fatty acids (Fig. 3G).

To further explore the mechanism of CO₂-mediated growth promotion in *A. muciniphila*, we tested short-chain fatty acids (formic acid, acetic acid, propionic acid) (Fig. S4), amino acids (all 20) (Fig. S5), culture supernatants (Fig. S6), and cell lysates (Fig. S7). None of them promoted *A. muciniphila* growth in CO₂ minus conditions. We tested the pH of the medium before and after culture, with no differences (Fig. S8). Considering that CO₂ can act as an electron acceptor for the growth of anaerobic microbes to generate ATP and promote cell growth [51], we found that adding electron acceptors such as nitrate, TMAO, and bicarbonate effectively stimulated *A. muciniphila* growth in the absence of CO₂, whereas adding pyruvate and sulfite did not. This suggested that CO₂ likely promotes *A. muciniphila* growth by serving as an electron acceptor and providing energy for growth initiation (Fig. 3H). Furthermore, CO₂ addition stimulated the expression of the probiotic genes Amuc_1100 and Amuc_1831 (Fig. 3I, J), while other reported Amuc proteins showed no significant difference (Amuc_1409, Amuc_1438, Amuc_2172, Fig. S9). These findings, along with transcriptomic and metabolomic data, indicate that CO₂ plays broader physiological regulatory roles in *A. muciniphila*.

### CIAs generating CO_2_ within the gut promoted the growth of *A. muciniphila* and induced weight loss in HFD mice

**Figure 4:**
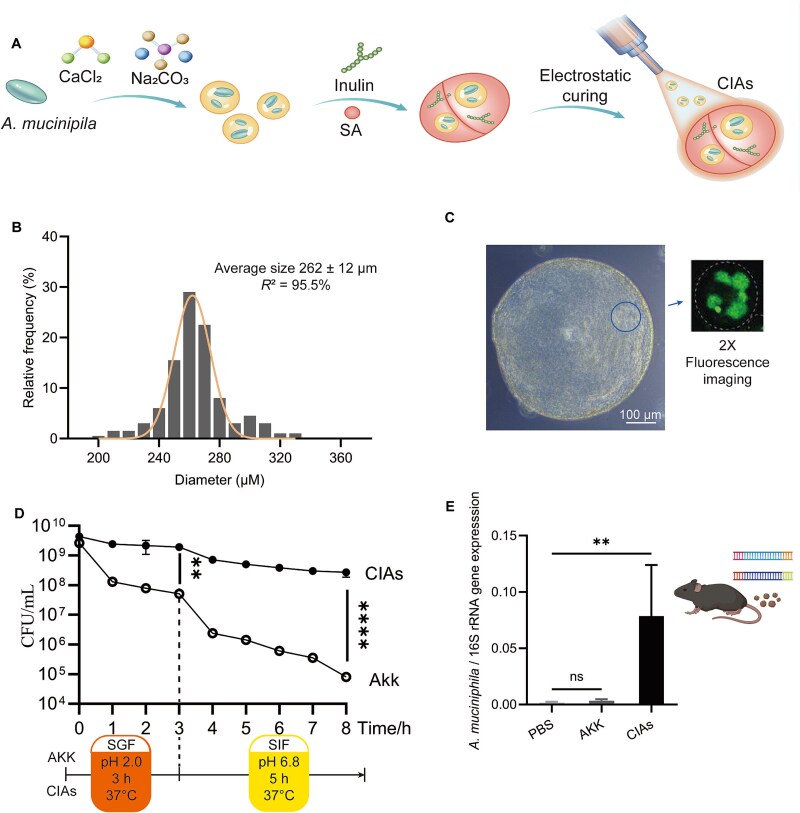
Preparation and evaluation of *A. muciniphila* live cell delivery system. (A) The preparation process of the *A. muciniphila* live bacteria delivery system (CIAs). Addition of calcium chloride, sodium carbonate, inulin, and sodium alginate (SA) to the *A. muciniphila* bacterial solution, followed by electrostatic curing. (B) The particle size distribution of CIAs. Average size is presented as the means ± SD. (C) Representative image of CIAs in bright field and fluorescence microscopy. Scale bar in bright field: 100 μm. Fluorescence microscopy is 2x to bright field. (D) CIAs significantly enhanced *A. muciniphila* resistance to simulated gastric fluid (SGF) or simulated intestinal fluid (SIF). (E) Survival of CIAs and *A. muciniphila* free cells in the mouse gastrointestinal tract 72 hours post-gavage. ^**^P < 0.01, ^***^P < 0.001, ns (no significance).

To leverage the CO₂-promoting effect on *A. muciniphila* growth, we designed a nanoencapsulation system containing **c**alcium carbonate, **i**nulin, ***A**. muciniphila*, and **s**odium alginate, termed CIAs. This system aims to enhance *A. muciniphila* growth and probiotic function in the gut via CO₂ production through microbial fermentation. Initially, *A. muciniphila* cells, CaCl₂, and Na₂CO₃ were mixed in a neutral pH 7 solution, resulting in CaCO₃ deposition on the surface of *A. muciniphila* cells. Subsequently, inulin and sodium alginate were added to the slightly acidic mixture. The binding of sodium alginate with calcium ions led to the formation of calcium alginate microspheres filled with inulin and CaCO₃. Electrospinning was employed to control the uniformity and stability of the microspheres (Fig. 4A). The diameter of the CIAs microsphere was 262 ± 11.9 μm (Fig. 4B), with the *A. muciniphila* cells uniformly distributed (Fig. 4C). The sodium alginate maintained a colloidal structure that helped *A. muciniphila* to resist the acidic stomach environment. And CIAs could tolerate digestive enzymes in simulated gastric/ intestinal fluid (Fig. 4D). Upon reaching the neutral pH of the intestine, the sodium alginate disintegrated, and the released inulin was metabolized by intestinal microbiota to produce SCFAs. SCFAs hydrolyzed the CaCO₃ on *A. muciniphila* cells, generating CO₂ and promoting *A. muciniphila* growth (Fig. 4E).

**Figure 5:**
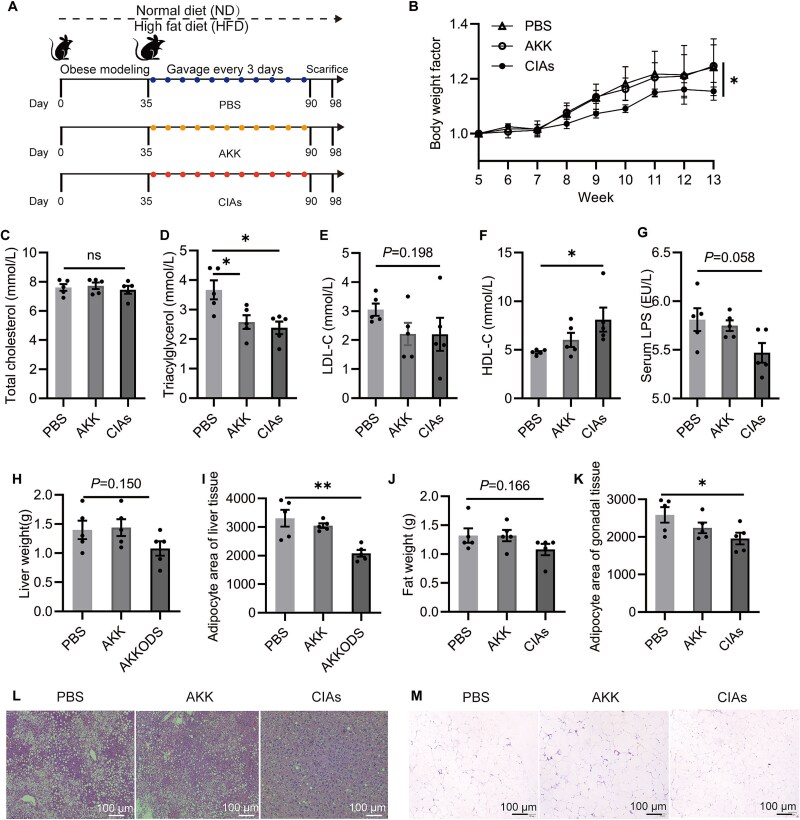
CIAs induce weight loss in high-fat diet mice model. (A) Schematic diagram showing various administration strategies of *A. muciniphila* and CIAs on HFD mice, with 6 mice per group. Mice were fed a normal diet (ND) or an HFD (60% fat). The obese mice were treated with CIAs or *A. muciniphila* strain (10^7^–10^8^ CFU per mouse) every 3 days from Day 35 to Day 90. (B) the proportion of body weight over time in each group. Blood-related indicators levels of total cholesterol (C), triacylglycerol (D), low-density lipoprotein cholesterol (LDL-C) (E), high-density lipoprotein cholesterol (HDL-C) (F), lipopolysaccharides (LPS) (G). Liver fat tissue (H) and gonadal adipose tissue (J) mass, and adipocyte area in liver (I) and gonadal (K) adipose tissue. Representative hematoxylin and eosin (H&E) staining of liver (L) and gonadal adipose tissue (M). Nuclei are stained in blue, while the extracellular matrix and cytoplasm are stained in red using H&E staining. ^*^P < 0.05, ^**^P < 0.01, ns (no significance).

We further evaluated the efficacy of the CIAs system in a high-fat diet mouse model (Fig. 5A). After 6 weeks of HFD/ND feeding, significant differences in body weights were produced between the two groups of mice (Fig. S10), and the HFD group mice were re-grouped for subsequent experiments. To eliminate the influence of inoculum size, a low inoculum (5 × 10^7^ CFU per mouse) of *A. muciniphila* was used. Compared to the PBS group (phosphate buffered saline, control group), this low inoculum of *A. muciniphila* did not result in significant weight loss (Fig. 5B), consistent with previous reports [52]. In contrast, CIAs significantly reduced body weight in mice (Fig. 5B). There was no significant difference in total cholesterol levels among groups (Fig. 5C). Additionally, CIAs showed significant differences to the PBS group in triacylglycerol (Fig. 5D), HDL-C (high-density lipoprotein cholesterol, Fig. 5F), and adipocyte area in liver (Fig. 5I) and fat tissue (Fig. 5K). Comparing the CIAs and PBS groups, LDL-C level (low-density lipoprotein cholesterol, Fig. 5E), serum LPS (lipopolysaccharides, Fig. 5G), liver weight (Fig. 5H), and fat weight (Fig. 5J) also showed a decreasing trend. CIAs also showed beneficial results on H&E staining of liver (Fig. 5L) and gonadal adipose (Fig. 5M) tissues.

These data indicate that the CIAs delivery system, designed based on the CO₂-dependent growth phenotype of *A. muciniphila*, significantly enhances the survival of *A. muciniphila* in mice and exhibits substantial probiotic effects.

### CO_2_ modulation alters gut microbiota composition and metabolic pathways

**Figure 6:**
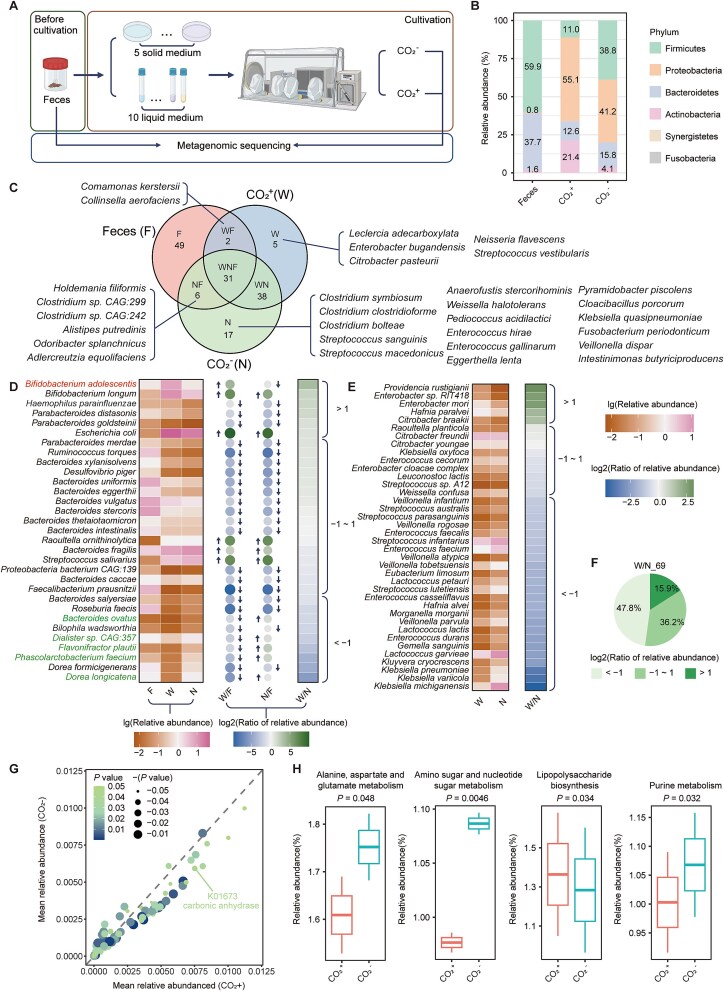
The impact of CO_2_ on the composition and function of human-derived gut microbiota. (A) Schematic diagram of the culturomics experiment. (B) Composition of species at the phylum level for the Feces, CO_2_^+^ group, and CO_2_^−^ group. (C) Overlap of species among the three groups. (D) Relative abundance of the 31 bacteria found in all three groups (left subfigure), the ratio of the relative abundance of the 31 bacteria in the CO_2_^+^ and CO_2_^−^ groups compared to the Feces (middle subfigure, upward arrow indicates log2(ratio of relative abundance) > 0, downward arrow indicates log2(ratio of relative abundance) < 0), and the ratio of the relative abundance between the CO_2_^+^ and CO_2_^−^ groups (right subfigure). (E) Relative abundance of the 38 bacteria found only in the CO_2_^+^ and CO_2_^−^ groups (left subfigure), and the ratio of relative abundance between the two groups (right subfigure). (F) Distribution of the ratio of relative abundance for the 69 bacteria common to the CO_2_^+^ and CO_2_^−^ groups. Significant differences in genes (G) and pathways (H) between the CO_2_^+^ and CO_2_^−^ groups.

To explore whether CO₂ affects other gut microbiota as it does on *A. muciniphila*, we collected fecal samples from five healthy adult volunteers, homogenized them, and inoculated them into five types of solid media and ten types of liquid media as descripted in methods. These samples were cultured in anaerobic chambers with and without CO₂. DNA was extracted from the initial fecal mixtures and the cultures, followed by metagenomic sequencing (Fig. 6A).

**Figure 7:**
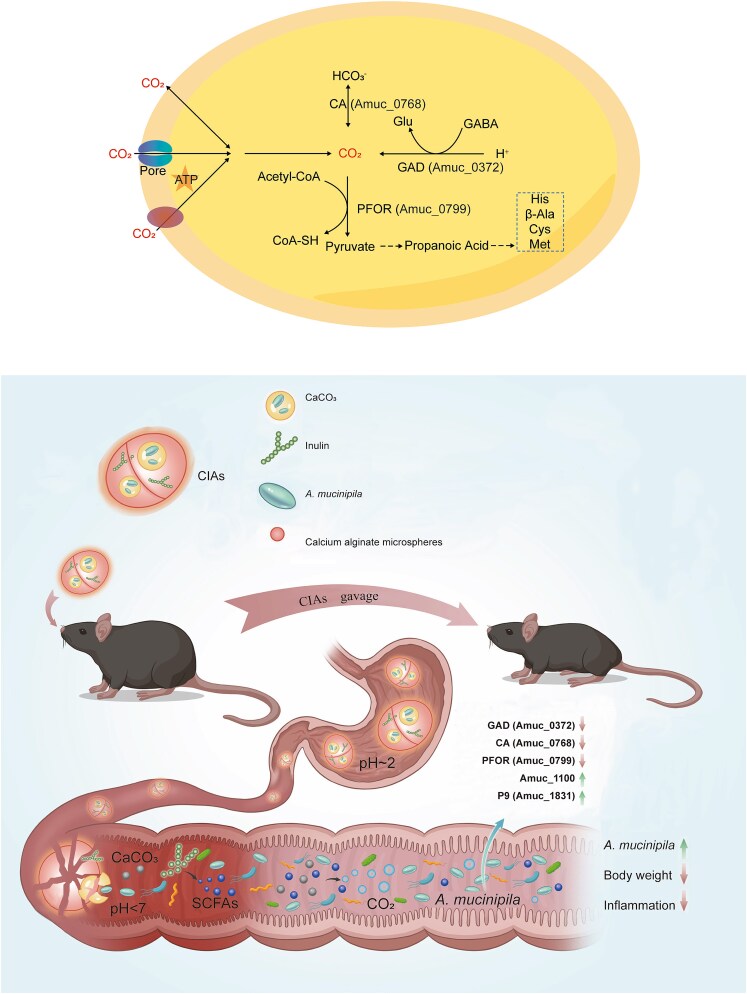
Schematic diagram of this research. CO_2_ involved metabolic processes in *A. muciniphila* and improved the obese mice.

Post-culturing, a substantial shift in the microbial composition was observed (Fig. 6B, C), consistent with previous report [53]. Our findings indicate that CO₂ significantly alters the structure of human fecal microbiota, with 76 and 92 different bacterial species identified under CO₂-supplemented and non-supplemented conditions, respectively (Fig. S11). To compare the differences between the fecal microbiota and the microbiota cultivated under CO_2_ and non-CO_2_ conditions, we performed a PCoA analysis (Fig. S12). From the figure, we can see that the distance between the two samples under different CO_2_ conditions for the same medium is smaller than the distance between those samples and the fecal samples, indicating that cultivation rather than CO_2_ was the main factor influencing the microbiota structure. This finding is consistent with our analysis at the phylum level of species composition. However, we did find that CO_2_ had a significant impact on certain bacteria. For instance, *Comamonas kerstersii* and *Collinsella aerofaciens* were only detected in the fecal and CO_2_+ groups (Fig. 6C), suggesting that CO_2_ is crucial for their growth. The abundance of *Bifidobacterium adolescentis* increased (log₂ ratio of relative abundance = 3.41) in the CO₂ group, while it decreased in the non-CO₂ group (log₂ ratio of relative abundance = −0.1). Conversely, *Bacteroides ovatus*, *Bilophila wadsworthia*, *Dialister* sp. CAG:357, *Flavonifractor plautii*, *Phascolarctobacterium faecium*, and *Dorea longicatena* exhibited opposite trends, underscoring the negative impact of CO₂ on these bacteria (Fig. 6D; Table S7). Compared to the non-CO₂ group, 15.9% of bacterial species exhibited a significant increase in relative abundance in the CO₂ group (log₂ ratio of relative abundance >1), including *Bifidobacterium adolescentis*, *Bifidobacterium longum*, *Providencia rustigianii*, *Parabacteroides goldsteinii*, *Enterobacter mori*, and *Escherichia coli* (Fig. 6D-F; Tables S7, S8). It is widely accepted that *B. adolescentis* and *B. longum* are well-known probiotics [54], suggesting that CO₂ could exert probiotic effects by modulating the gut microbiota.

A metabolic pathway enrichment analysis revealed that lipopolysaccharide biosynthesis (*P* = 0.034) was significantly enriched in the CO₂ group, whereas pathways such as alanine, aspartate, and glutamate metabolism (*P* = 0.048), amino sugar and nucleotide sugar metabolism (*P* = 0.0046), and purine metabolism (*P* = 0.032) were significantly reduced. These pathway alterations could be related to the mechanisms by which CO₂ promotes bacterial growth and regulates bacterial physiological functions (Fig. 6H).

Furthermore, the CA gene was identified among the significantly enriched gut microbiota genes in the CO₂ group, suggesting that the ability of CA to facilitate CO₂ fixation may confer a growth advantage to bacteria in CO₂-rich conditions (Fig. 6G; Table S9).

## Discussion

*A. muciniphila* is an indigenous gut bacterium residing in the intestinal mucosal layer, and it is closely associated with host health. Traditional probiotics such as *Bifidobacterium* and *Lactobacillus* have a long history of widespread human consumption [55]. *A. muciniphila* supplementation is positioned as a next-generation probiotic, given that it shows promise in treating medical conditions like obesity [56], depression, and Alzheimer’s disease [57]. Despite these advantages, widespread clinical applications of *A. muciniphila* remain limited. A clinical study [35] demonstrated that significant weight loss effects are achievable only with high doses of *A. muciniphila*, while related research [58] observed an increase in *A. muciniphila* abundance in conditions such as Crohn’s disease and ulcerative colitis. The mucus-degrading properties of *A. muciniphila* are beneficial for promoting mucosal turnover in individuals with intact mucosal barriers. However, in those with compromised intestinal epithelial integrity, mucus degradation may expose damaged intestinal tissues to the gut microbial environment, potentially allowing inflammatory factors like LPS to enter the bloodstream and exacerbate health issues [59]. Given the current inadequacy of extensive clinical research and possible risks of high-dose supplementation, promoting *A. muciniphila* growth and enhancing its efficacy through prebiotics represents a safer and more prudent approach. Preliminary data suggest that compounds such as cobalt-amino complexes, vitamin B12 [60], and cranberry extracts [61] can promote *A. muciniphila* growth in both in vitro and in vivo research.

Here, we discovered that CO₂ can rapidly stimulate *A. muciniphila* growth. The effects of self-generated and supplemental CO_2_ on *A. muciniphila* show similar effects in growth (Fig. S13) and expression of *A. muciniphila*’s probiotic proteins (Fig. S14). By leveraging the *A. muciniphila* CO₂-dependent growth phenotype, we designed a novel live bacterial delivery system, CIAs, incorporating calcium alginate to protect *A. muciniphila* from gastric acidity. Inulin, through microbial metabolism, generates beneficial SCFAs, while hydrogen ions neutralize the carbonate ions in calcium carbonate, producing CO₂ to promote *A. muciniphila* rapid growth. Our designed CIAs system effectively facilitated *A. muciniphila* proliferation in the intestines of mice fed HFD, resulting in a significant weight reduction (Fig. 5). These findings propose a therapeutic strategy utilizing live bacterial treatments to promote *A. muciniphila* growth, thereby harnessing its probiotic effects in treating diseases. To achieve the goal of widespread clinical application of next-generation probiotics such as *A. muciniphila*, the large-scale production of viable *A. muciniphila* through modern fermentation techniques is imperative. Based on the *A. muciniphila* CO₂-dependent growth mechanism, we found that simple NaHCO₃ addition can effectively promote *A. muciniphila* growth, suggesting a straight forward and efficient *A. muciniphila* cultivation process (Fig. 3H, Fig. S15).

From the transcriptome and proteome data, we identified 384 differentially expressed genes and 470 differentially expressed proteins (Supplementary Tables S1 and S2). The proteins encoded by AMUC_RS02075 (Amuc_0372) glutamate decarboxylase (GAD) and AMUC_RS04140 (Amuc_0768) γ-carbonic anhydrase family protein (CA), which are directly related to CO_2_, are significantly upregulated after CO_2_ dissipation. This suggests that *A. muciniphila* may adapt to CO_2_ fluctuations by regulating the expression of these key enzymes. Gene ontology (GO) enrichment analysis showed that the upregulated genes are primarily enriched in terms such as ATP binding (GO:0005524) and ATP hydrolysis activity (GO:0016887). This indicates that under different CO_2_ conditions, *A. muciniphila* may adjust its energy metabolism pathways to adapt to environmental changes. The upregulated genes also involve transmembrane transport (GO:0055085) and membrane (GO:0016020) functions, which may help *A. muciniphila* adjust the transport of substances and membrane permeability during CO_2_ fluctuations, thereby maintaining internal environmental stability.

Moreover, electron acceptor addition experiment indicates that electron acceptors such as nitrate and trimethylamine N-oxide (TMAO) mimic CO₂'s effects, suggesting their role in facilitating the complete electron transport chain necessary for *A. muciniphila* rapid growth and energy acquisition. TMAO, derived from host gut microbial metabolism of choline, adversely affects cardiovascular and neurological diseases [62]. In conditions of gut inflammation, host-generated nitric oxide (NO) can be converted to nitrate, which is utilized by certain Enterobacteriaceae like *E. coli* for rapid growth, exacerbating disease progression [24]. Our findings suggest that *A. muciniphila* can sense and initiate rapid growth in response to these substances, potentially implicating *A. muciniphila* in combating relevant diseases. The differential effects of electron acceptors on *A. muciniphila* growth likely arise from the distinct biochemical pathways involved in their reduction. Nitrate, TMAO, and bicarbonate may be more efficiently utilized by *A. muciniphila*. Future investigations of the specific enzymes and metabolic pathways involved are needed to elucidate these mechanisms.

Furthermore, the significant regulatory effect of CO_2_ on other gut microbiota, enriching metabolic pathways such as lipopolysaccharide biosynthesis, underscores its potential to modulate host immune systems through gut microbiota regulation. The abundance of *Bifidobacterium (B. adolescentis* and *B. longum)* increased in the CO_2_+ group (Fig. 6D), and related studies have shown the importance of CO_2_ for colony development in *Bifidobacterium* species. These species have difficulty forming colonies in a 100% N_2_ gas environment, but develop well when 1% CO_2_ is added [63]. Additionally, previous research has reported under anaerobic conditions, when the CO_2_ concentration reaches 20%, it can significantly promote the growth of *B. longum* [64].

We also observed a significant enrichment of the gene encoding CA, suggesting its crucial role in CO₂ utilization by bacteria. CA, a zinc-centered metalloenzyme, plays pivotal roles in maintaining acid–base balance, transporting CO₂ and bicarbonate, and various biosynthetic reactions across mammals, plants, and microorganisms [65]. CA family proteins have emerged as potential drug targets in cancer, diuretic, anti-glaucoma, and anti-epileptic therapies [65]. Our research highlights that modulating CA function can regulate gut microbiota growth and activity, potentially improving human health outcomes.

Related research have showed that gut CO₂ concentrations reflect ancient atmospheric levels [66]. As soil microorganisms utilize CA to capture atmospheric CO₂ for soil carbon sequestration [67], insights from gut microbiota CA research could also inform geological microbial CO₂ studies.

However, although the positive effects of CO₂ on *A. muciniphila* rapid growth and probiotic protein gene expressions were elucidated (Fig. 7), the molecular mechanisms underlying these impacts on *A. muciniphila* remain unclear. In addition, this study only evaluated the safety and therapeutic effects of the CIAs system in a mouse model. For clinical applications, comprehensive safety assessments and clinical trials are appreciated.

## Supplementary Material

Supplemental_Figures_wraf034

Supplemental_Tables_wraf034

## Data Availability

All sequencing data have been uploaded to CNCB (China National Center for Bioinformation, https://www.cncb.ac.cn). The RNA-seq project number is PRJCA034716, the proteome project number is PRJCA034669, and the culture group project number is PRJCA034716. The data that support the findings of this study are available from the corresponding author upon reasonable request.
