# Supplementary material for: Carbon dioxide enhances Akkermansia muciniphila fitness and anti-obesity efficacy in high-fat diet mice
Source: ISME J. 2025 Feb 23;19(1):wraf034. doi: 10.1093/ismejo/wraf034 (PMC11931619; doi:10.1093/ismejo/wraf034)
Supplement: Supplemental_Figures_wraf034 [file supplemental_figures_wraf034.pdf]

Supplementary Figures

Figure S1

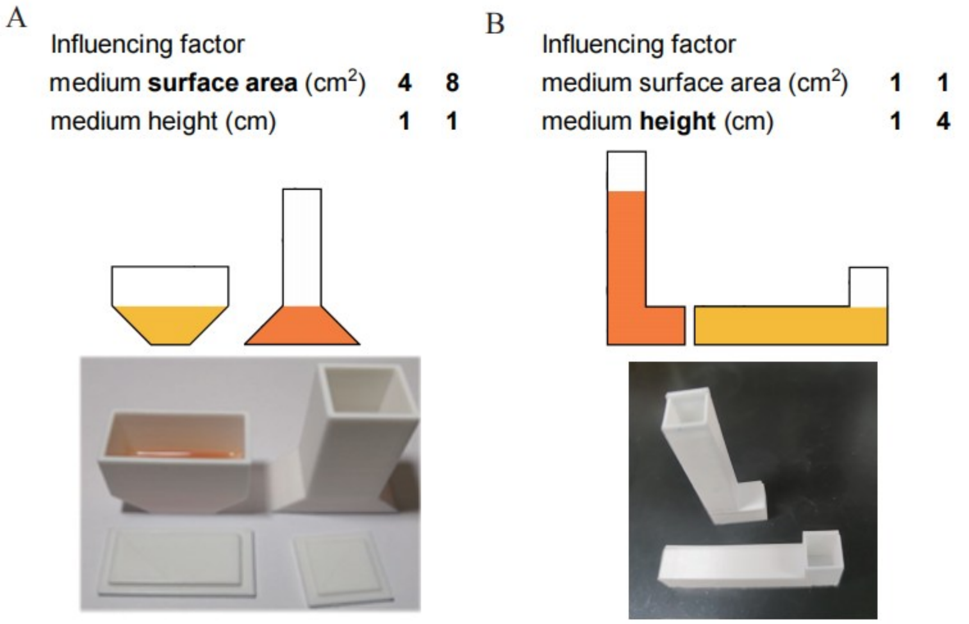

Figure S1 3D printed models and actual images of the surface area (A) or height (B) differences of the culture medium.

Figure S2

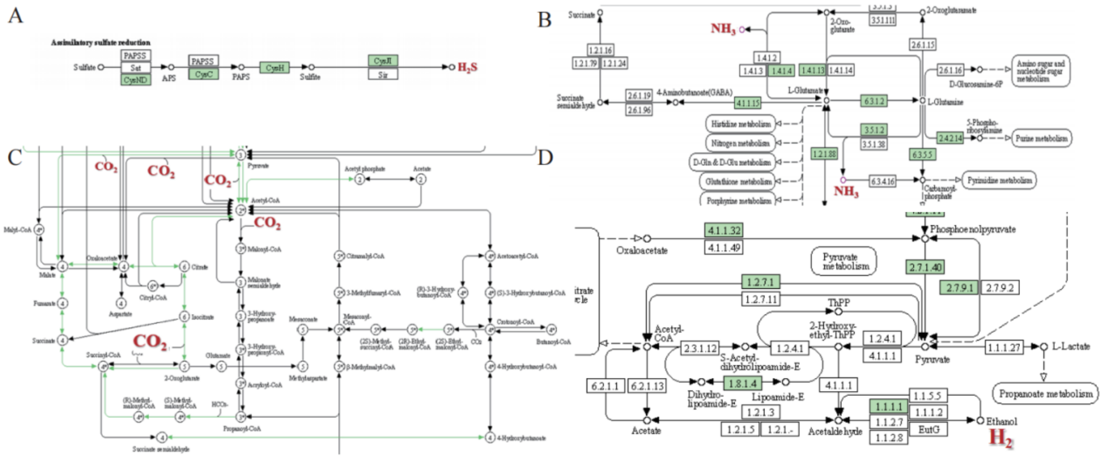

Figure S2 Four small molecule gas components (A: H_2S, B: NH_3, C: CO_2, D: H_2) produced by AKK obtained through GSMM analysis and KEGG alignment.

Figure S3

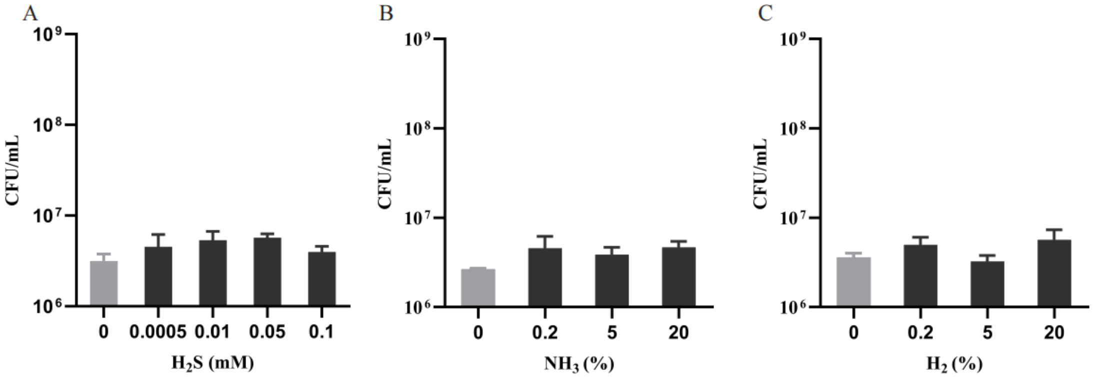

Figure S3 Effects of different concentrations of small molecule gas components (A: H_2S, B: NH_3, C: H_2) on the growth of AKK. **$p<0.01$, ***$p<0.001$.

Figure S4

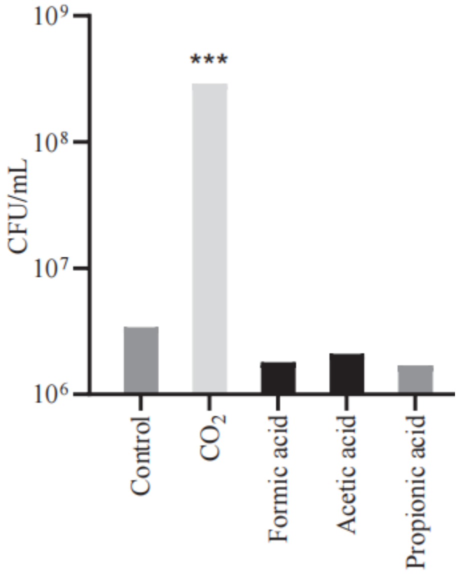

Figure S7 Results of short-chain fatty acids (SCFAs) addition on AKK growth. ***$p<0.001$, unmarked p-values are not significance.

Figure S5

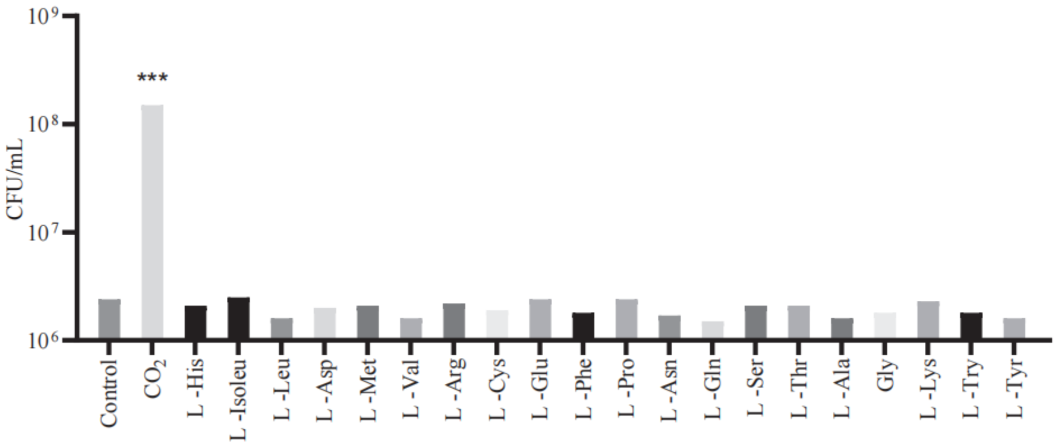

Figure S5 Results of amino acids supplementation on AKK growth. ***p<0.001, unmarked p-values are not significance.

Figure S6

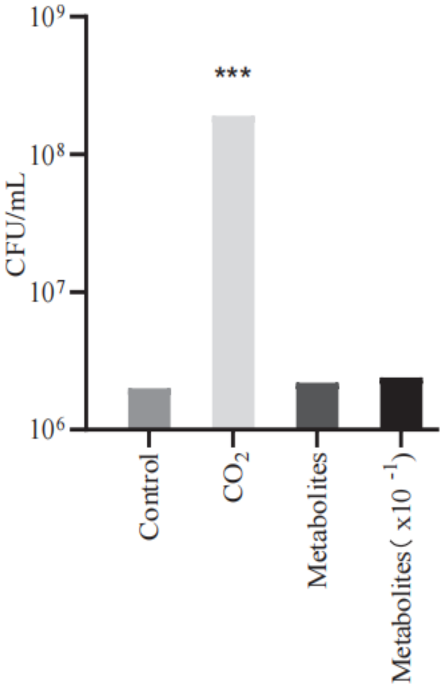

Figure S6 Results of different concentrations of metabolites addition on AKK growth. ***p<0.001, unmarked p-values are not significance.

Figure S7

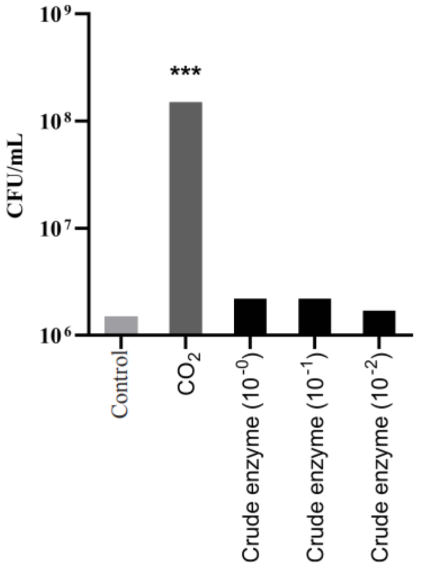

Figure S7 Results of different concentrations of crude enzyme supplementation on AKK growth. ***$p<0.001$, unmarked p-values are not significance.

Figure S8

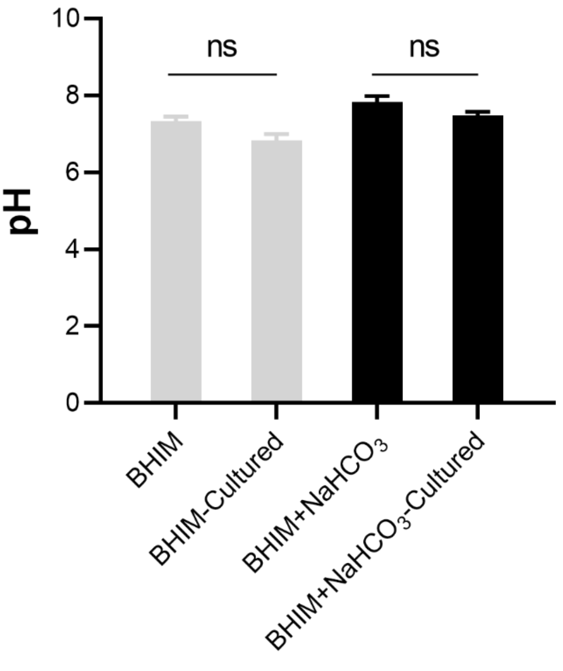

Fig. S8 pH values before and after culture. ns indicates no significantly difference in results at $p > 0.05$.

Figure S9

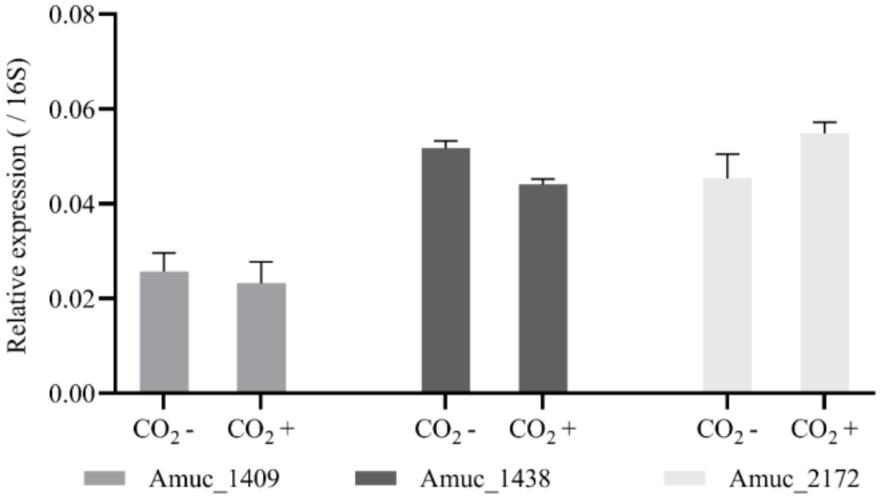

Figure S9 Results of relative expression of Amuc_1409, Amuc1438, Amuc_2172 under the difference of CO_2.

Figure S10

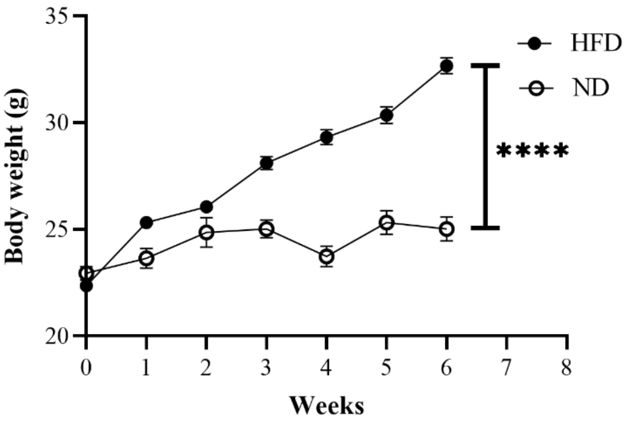

Fig. S10 Weight changes during the obesity mouse modeling process. In the 6-week obesity mouse model, we recorded the weight changes of mice in the high-fat diet modeling group and the normal diet control group. The obesity model was considered successful and ready for subsequent experiments when the body weight of mice in the high-fat diet group exceeded 30% of that in the normal diet control group.

Figure S11

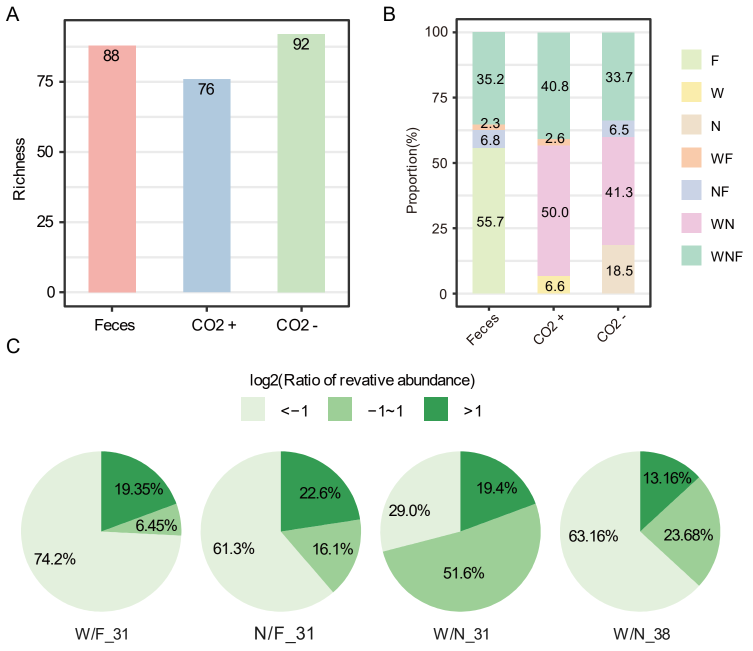

Figure S11 A shows the number of species detected in the Feces, CO_2+ and CO_2- groups. B shows the proportions of 7 categories of species in each group. C shows the distribution of relative abundance ratios of species.

Figure S12

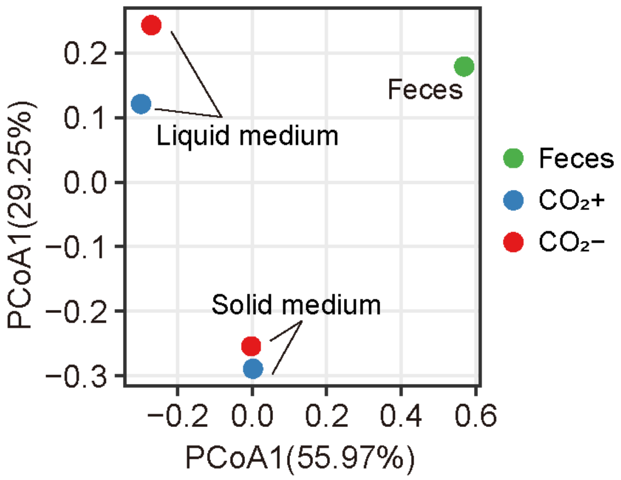

Fig. S12 PCoA analysis of the fecal microbiota and the microbiota cultivated under CO_2-/+ conditions.

Figure S13

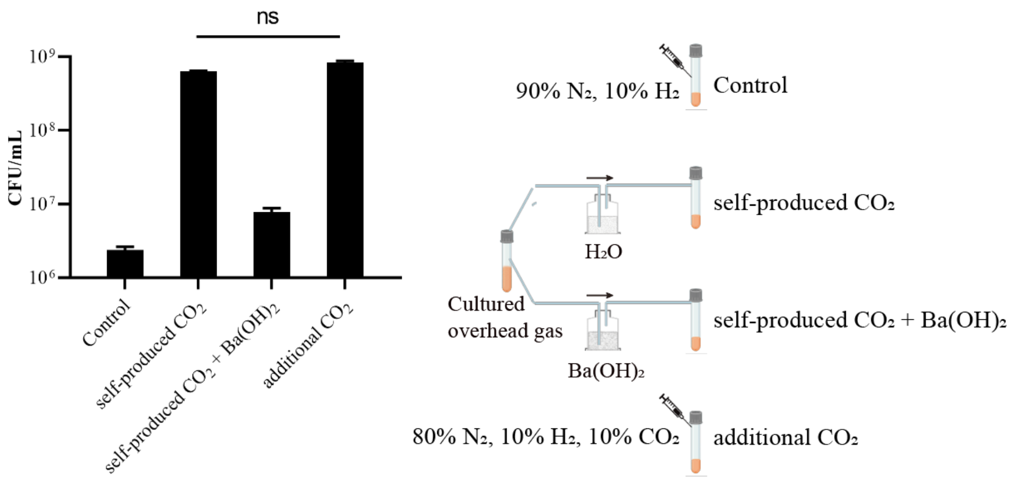

Fig. S13 Effects of self-produced and additional CO_2 on *A. muciniphila* growth (Left, growth results, Right, schematic diagram of the experiment). ns, no significance.

Figure S14

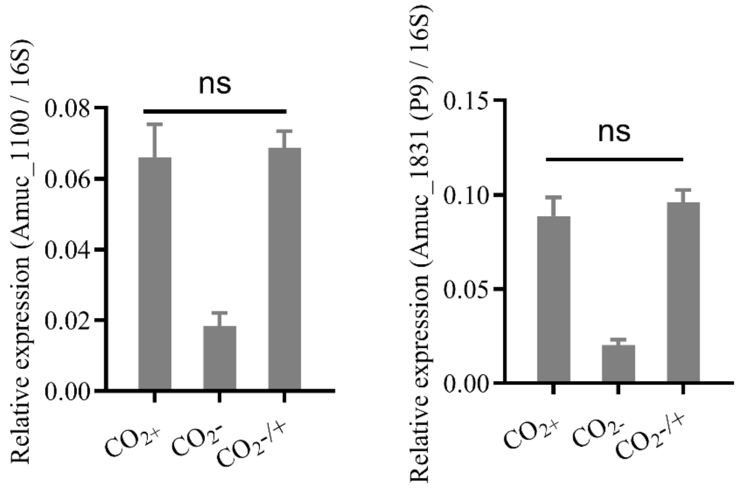

Fig. S14 CO_2 produced by *A. muciniphila* themselves and CO_2 supplemented induced the expression of Amuc_1100 (left) and Amuc_1831 (right) in *A. muciniphila*. Group CO_2- dissipated CO_2 by stirring, group CO_2+ did not stir, group CO_2-/+ stirred for 10 min then added additional gas CO_2. ns, no significance.

Figure S15

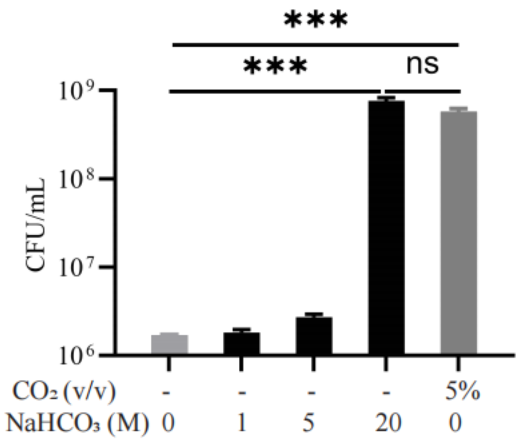

Figure S15 $NaHCO_3$ and CO_2 have similar effects in promoting rapid growth of AKK. ***p<0.001, ns = no significance.

Figure S16

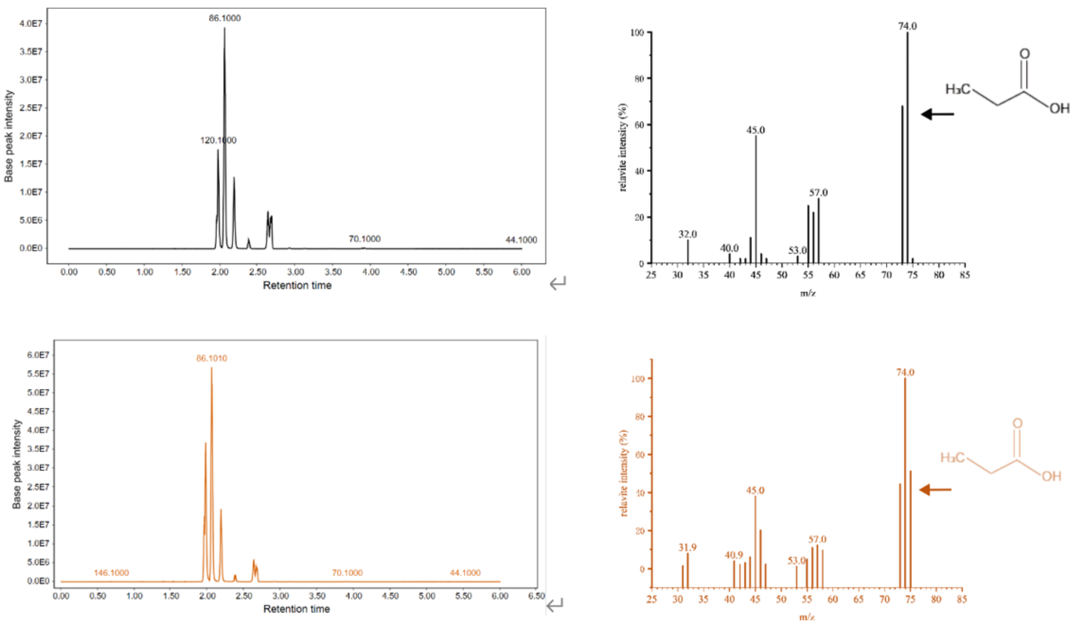

Figure S16 GC-MS images of detected ^{13}C-labeled SCFAs by adding $^{13}CO_2$ to the *A. muciniphila* culture environment. The left panel shows the total ion chromatogram, with the upper left indicating the ^{12}C-labeled control group and the lower left representing the ^{13}C-labeled experimental group. The right panel displays the results from comparing the sample peaks with those of standard compounds. The two groups on the right show the propionic acid detection peaks, with the upper showing the ^{12}C-labeled control group and the lower showing the ^{13}C-labeled experimental group.

Figure S17

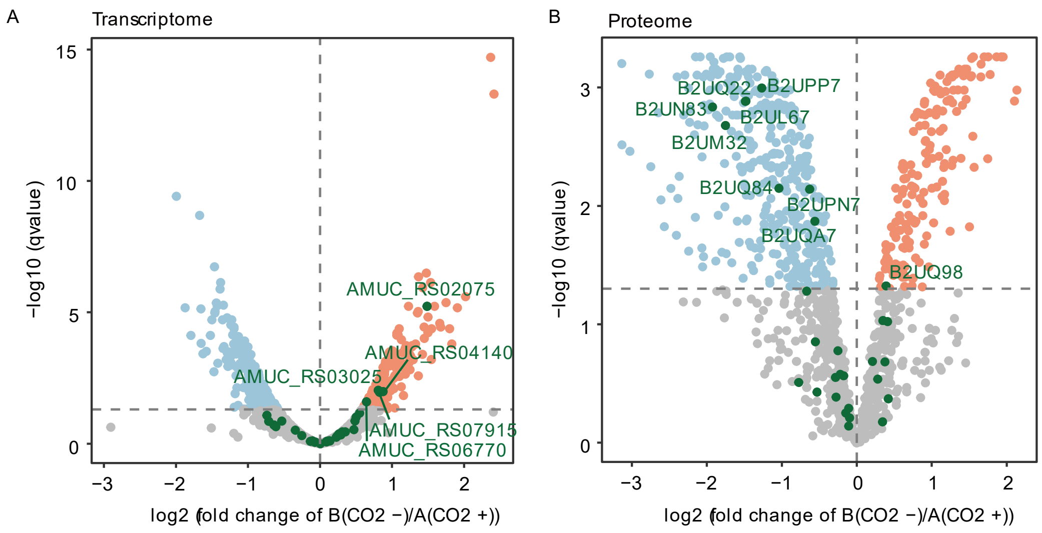

Figure S17 Transcriptome data (A) and proteome data (B) volcano plot, and green points represent CO_2-related genes or proteins.

Figure S18

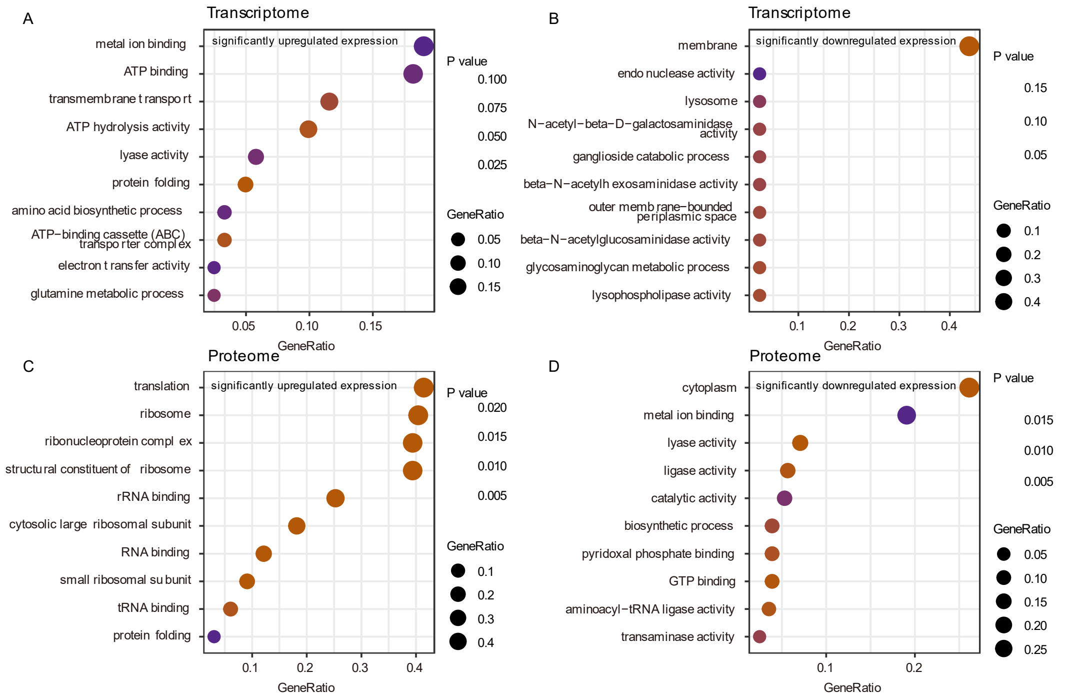

Figure S18 GO enrichment results of differentially expressed genes and corresponding genes of differentially expressed proteins. A, GO enrichment results of significantly upregulated genes in transcriptome; B, GO enrichment results of significantly downregulated genes in transcriptome; C, GO enrichment results of corresponding genes of significantly upregulated proteins in proteome; D, GO enrichment results of corresponding genes of significantly downregulated proteins in proteome.

Figure S19

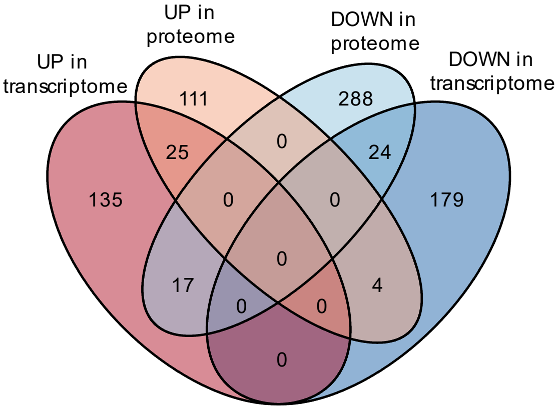

Figure S19 Overlap between differentially expressed genes obtained from transcriptome data and differentially expressed proteins obtained from proteome data.
